# Assessment of the Socioeconomic Status and Analysis of the Factors Motivating Patients to Apply for Prosthetic Treatment by Students of Dentistry at the Poznań University of Medical Sciences

**DOI:** 10.3390/ijerph19095703

**Published:** 2022-05-07

**Authors:** Dominik Medyński, Tomasz Gredes, Mariusz Glapiński, Damian Dudek, Beniamin Oskar Grabarek, Ewa Niewiadomska, Agata Czajka-Jakubowska, Agnieszka Przystańska

**Affiliations:** 1 Department of Prosthodontics and Gerostomatology, Poznań University of Medical Sciences, 60-812 Poznan, Poland; 2 Department of Orthodontics and Temporomandibular Disorders, Poznań University of Medical Sciences, 60-812 Poznan, Poland; tomasz.gredes@ump.edu.pl (T.G.); a.czajka-jakubowska@ump.edu.pl (A.C.-J.); 3 Department of Orthodontics, Carl Gustav Carus Campus, Technische Universität Dresden, 01069 Dresnen, Germany; 4 Department of Anatomy, Poznań University of Medical Sciences, 60-781 Poznan, Poland; mglapinski@ump.edu.pl (M.G.); aprzyst@ump.edu.pl (A.P.); 5 Artmedical Oral Surgery, Szosa Chelimnska 166, 87-100 Torun, Poland; damiandudek@op.pl; 6 Department of Histology, Cytophysiology and Embryology, Faculty of Medicine in Zabrze, The University of Technology in Katowice, Academy of Silesia, 41-800 Zabrze, Poland; bgrabarek7@gmail.com; 7 Department of Epidemiology and Biostatistics, School of Health Sciences in Bytom, Medical University of Silesia, 40-055 Katowice, Poland; eniewiadomska@sum.edu.pl

**Keywords:** socioeconomic factors, motivation, prosthodontics, students, dental

## Abstract

This study aimed to determine the motives for undertaking prosthetic treatment at the Prosthetics Clinic of the Poznan University of Medical Sciences (PUMS), pursued by fifth- and sixth-year medicine and dentistry students. The survey consisted of 18 questions, of which 1 to 11 concerned general patient data, while questions 12 to 18 concerned prosthetic treatment. The only open question in the questionnaire was one regarding the motives for the decision to receive treatment. The study group consisted of 153 patients (102 women—66.67% and 51 men—33.33%) of the Prosthetic Clinic. After collecting a total of 200 questionnaires, rejecting 47 (23.50%) due to the lack of answers to all questions (other than question 18), a total of 153 completed (76.50%) questionnaires were obtained. The main motivating factors for patients to undertake prosthetic treatment are functional and aesthetic considerations. In addition, it turned out, that the authority of the university unit is of greater importance than financial issues. Additionally, some patients undergo treatment by students because they can see positive aspects in it, both for them and for students. Hence, the evaluation of the treatment provided by students, as well as their communication skills and their attitude, are rated very highly, although, again, the evaluation was influenced by the education level.

## 1. Introduction

The most common motivating factors for patients to undertake prosthetic treatment are the improvement of function by replacing missing teeth or repairing already worn restorations, aesthetic improvements, or referral by a doctor of another specialization. In dental treatment, there is also a negative motivating factor—anxiety called dentophobia. This is a major problem for both the doctor and the patient as the person who is trained to help patients and alleviate their suffering is perceived to be the perpetrator of this pain. Shah et al. found that a large number of those surveyed avoided a visit to the dentist until they required intervention [1]. In turn, in the study conducted by Hällström and Halling, a strong fear of the dentist was found in 13.4% of the studied population. It is common to see patients who openly admit that, for a very long period of their life, they avoided the dentist out of fear, but now they want to undergo treatment that will restore the neglected function and appearance [2]. This only happens when the positive motivating factor is stronger than the negative one [3]. The second reason for not starting treatment is the lack of awareness of its need. Parlani et al. conducted an educational and motivational program on a group of 227 patients. The rise in prosthetic needs after the program increased by 10% in relation to the situation beforehand. Motivating patients by medical personnel is a very important component of prevention [4].

Due to the specificity of oral diseases, except in the case of painful situations or accidents, dental procedures rarely qualify as emergencies. The research of Pavi et al. indicated that for only 32.6% of respondents, prophylaxis was the main motivation for using the dentist’s services [5]. In turn, in the study by Gârdan and Gârdan [6], 17% of respondents were convinced of the need to visit only when symptoms appeared. Patients entering treatment are often motivated by the desire to improve their quality of life and psychosocial well-being, and to improve self-confidence and self-esteem, in addition to the obvious improvement in appearance and function [7,8].

It turns out that the self-assessment of the need for prosthetic treatment may depend on the effects of tooth loss, and these effects may have an aesthetic, functional, psychological, and social dimension. Anterior localization of the missing tooth increases the motivation for immediate treatment [9,10]

On the other hand, not all patients with missing teeth treat this condition as harmful, and women see a greater impact of tooth loss on quality of life than men [11,12].

Factors influencing treatment decisions can be very different, including age and gender of patients; number and location of missing teeth; impaired function and discomfort; in addition to social, cultural, and financial factors [13,14]. Pavi et al. [5] also add income; education level; place of residence; and health insurance. Trovik et al. [15] hypothesized that the need for tooth replacement is greater at the time of tooth loss.

The practical, independent participation in the procedures with which the doctor will have to deal with in everyday clinical practice is important for the development of the skills of future doctors, including clinicians. Abdulghani et al. [16] evaluated the attitude of patients towards care and treatment by medical students in two hospitals in Riyadh Saudi Arabia. Out of 492 respondents, 399 (81%) felt that students’ general appearance and manner were important to their willingness to have them participate in their care, and 315 (64%) would not object to the presence of medical students during physical examinations. In turn, 310 people (63%) preferred to be informed about students’ involvement beforehand. On the other hand, in a study completed by Graber et al. [17], it was shown that patients were unwilling for medical students to perform any kind of medical procedures during the patients’ stay in the Hospital Emergency Department. Patient beliefs were independent of age, gender, or insurance status. Nevertheless, an important factor determining the attitude of future doctors towards patients and vice versa is the development of social competencies. As was shown by the analysis of literature conducted by Leadbeatter et al. [18], social determinants of health and the development of social competencies among students of the medical and dental fields of study are covered and developed at an insufficient level. This was further confirmed by a study conducted by Holden et al. [19]. However, Mroczek et al. [20] showed that social competencies are mostly developed during the years of professional work. Therefore, it seems that patient trust in doctors, and the use of their services, may be expanded by the relationship between medical student and patient where important factors are knowledge, skills, and social competencies [17,18,19,20].

This study aimed to determine the motives for undertaking prosthetic treatment at the Prosthetics Clinic of the Poznan University of Medical Sciences, pursued by fifth- and sixth-year medicine and dentistry students.

## 2. Materials and Methods

### 2.1. Ethics

The questionnaire, which in the opinion of the Bioethics Committee operating at the Poznan University of Medical Sciences is not a medical experiment and does not require the consent of the committee (decision of 18 November 2021), was conducted entirely at the Dental Prosthetics Clinic of the Poznan University of Medical Sciences (PUMS), which is located on the premises of the University Center for Dentistry and Specialist Medicine (UCSiMS) at 70 Bukowska Street in Poznan.

### 2.2. Questionnaire

The presented study was conducted in the period from the beginning of December 2020 to the end of May 2021. Each person received a printed questionnaire in Polish (Supplementary Materials) and oral information about the anonymity and purpose of the study. This information was also included in the header of the survey. Additionally, the patient received a brief oral instruction on how to complete the form.

The survey consisted of 18 questions, of which 1 to 11 concerned general patient data, while questions 12 to 18 concerned prosthetic treatment. The only open question in the questionnaire regarding the motives for the decision to receive treatment was answered by 83 people (54.24% of the respondents). Due to the nature of the question, it was common for one person to provide multiple answers. Afterwards, the responses were grouped into the following sets: quick admission date; on the recommendation of relatives/friends; they have been treated here for a long time; low costs; treatment quality/professionalism; student education; pleasant atmosphere; diligence and gentleness; other responses that could not be classified together.

### 2.3. Characteristics of the Study Participants

Due to the place of research, it can be assumed with a high probability that people treated at the Clinic of Dental Prosthetics at the Medical University of Karol Marcinkowski in Poznań are residents of Poznań or neighboring towns in the Greater Poland Voivodeship. According to the data published by the Central Statistical Office (GSO) in 2020, the number of inhabitants of the Greater Poland Voivodeship was 3,496,500 [21]. The number of participants in the study was determined using the statistical tool available at https://www.naukowiec.org/dobor.html (accessed on 20 April 2022) [22]. For a population of 3,496,500 inhabitants, the maximum error value was estimated at 7%. Therefore, assuming a *p*-value < 0.05, the required number of respondents in the study is 180. In turn, for a population of 38,350,000 inhabitants, the maximum error value was estimated at 7%, and the required number of respondents in the study was 196 (*p* < 0.05). Our study included 200 participants.

After collecting a total of 200 questionnaires, rejecting 47 (23.5%) due to the lack of answers to all questions (other than question 18), a total of 153 (76.5%) completed questionnaires was obtained.

The study group consisted of 153 patients (102 women—66.67% and 51 men—33.33%) of the Prosthetic Clinic, who were patients who had been previously verified in terms of the possibility of receiving treatment as part of student clinical exercises, taking place in the fourth and fifth years of medical-dental studies, as well as patients referred by the main reception to qualify for student exercise treatment. Patients were verified and qualified by the doctors on duty at the clinic. Therefore, the study included both first-time patients and those whose treatment was previously planned or started.

The average age of the patients was 58.35 ± 13.17 years old, ranging from 24 to 84 years old, of which two were in the range of 20–30 years old. There were 15 patients in the age range of 31–40 years old. The second-largest group of respondents was 41–50 years of age, consisting of 32 people. In the 51–60-year-old age range, there were 25 patients. The most numerous groups were people aged 61–70, consisting of 46 people. The third-largest group was 71–80 years of age, including 31 patients. There were two people in the oldest age group of 81–90 years old. The largest group, consisting of as many as 80 people, were retirees and pensioners.

### 2.4. Price List for Prosthetic Services

The price lists available on the websites of twelve offices were analyzed, beginning with those that are considered expensive and reputable on the market and ending with less known ones. Commercial prices of these services, performed at UCSiMS, and also in force at the PUMS Prosthetics Clinic, were factored in as well. Commercial prices are the amounts that the patient pays for the service provided by the doctor–employee. At the time when the procedure was performed, the didactic prices applied.

### 2.5. Statistical Analysis

A database was created using the collected information and subjected to statistical analysis. Analysis of quantitative variables (i.e., expressed in number) was conducted by calculating the median (Me), and quartiles (Q1—down quartile, Q3—upper quartile). Analysis of qualitative (i.e., non-numeric) variables was conducted by calculating the number and percentage of occurrences of each value.

A comparison of the quantitative variable values in the two groups was performed through the Mann–Whitney U test. A comparison of the qualitative variable values in three and more groups was performed using the Kruskal-Wallis test. Following the detection of statistically significant differences, post-hoc analysis using Dunn’s test was conducted to identify statistically significantly different groups. A significance level of 0.05 was adopted in the analysis. The analysis was performed in the R program, version 4.1.0.

## 3. Results

### 3.1. The Socioeconomic Status of the Respondents

Thirty-four people were blue-collar workers (22.2%) and 30 people were white-collar workers (19.61%), whereas 9 people were unemployed (5.88%). Summing up the latter with retirees and pensioners, it turns out that as many as 80 people (52.29%) out of the 153 respondents were not working in any capacity. Among the people who indicated professional activity—64 people worked outside the health service (87.50%), while 7 people worked in this sector (10.94%). One person did not answer this question (1.56%).

Ninety-three people (60.78%) indicated their place of residence as a city with over a 100,000 residents. The second largest group were inhabitants of a village/small town in the vicinity of a large city—21 people (14%). The next largest groups were people living in a village and a town between 10–50,000 inhabitants, with 11 respondents in each group (7.19%). The same result also applied to the next two groups: people living in a town of between 5–10,000 residents (4.58%); and a town between 50–100,000 residents (4.58%), with 7 people in each group. The smallest group of respondents were residents of a town of up to 5000 inhabitants, with 1 person (0.65%). Two people did not answer this question (1.31%).

Just over half of the respondents—78 people (53.24%), were married. The next largest group were divorced people, with 25 respondents (17.12%). Next, 23 people were widowers and widows (15.75%). Twenty of the respondents (13.70%) were bachelors and maidens. The smallest group was people who defined their marital status as single—7 people.

Most respondents—103 people (67.32%), had adult children. Twenty people (13.07%) had no children, and 16 people (10.46%) had young children. Among all the surveyed people, 14 respondents (9.15%) mentioned having both young and adult children.

Among the survey-takers, the greatest number of respondents described their financial status as average—73 people (47.71%), whereas 46 people (30.07%) described their financial status as good. The group of patients describing their financial status as bad consisted of 30 people (19.61%). Only 2 people (1.31%) assessed their status as very good, and exactly the same number of respondents did not answer this question.

A monthly income of up to 2000 Polish zloty (PLN) per person was declared by 86 people (56.21%). In the income range of 2000 to 5000 PLN per person, there were 58 respondents (37.91%). The smallest group were people with an income above 5000 PLN per person—3 people (1.96%). Six people (3.92%) did not answer this question.

Almost three-quarters of the respondents—112 people (73.20%), declared that they did not have savings. Two groups had 14 people (9.15%): those declaring having cash savings above 10,000 PLN; those declaring having savings in the form of real estate. Nine people (5.88%) declared having savings in the form of a deposit, and 1 person (0.65%) marked the answer as “other”. None of the respondents (0%) declared that they had savings in the form of shares or art.

### 3.2. Relationship between the Need to Replace Old Prostheses and Sociodemographic Data

Firstly, whether and how different socioeconomic conditions of the respondents affect the need to replace old prostheses with new ones was assessed (Table 1). The need to replace the old prostheses was significantly more important for people aged 70+ than for people aged up to 40 and between 41–50 (*p* < 0.05); and for people aged 51–60 and 61–70 than for people aged up to 40 years old (*p* < 0.05); for the economically inactive than for the mentally employed (*p* < 0.05); for widows and widowers than for the other respondents (*p* < 0.05); for those married than for those divorced (*p* < 0.05); for those with adult children and those with young children (*p* < 0.05); for people in an average financial situation than for people in a good or very good condition (*p* < 0.05); for those in need of a complete denture than for those in need of a partial or permanent denture (*p* < 0.05).

### 3.3. Reasons for Undertaking Prosthetic Treatment

The evaluation of the factors motivating patients to undertake prosthetic treatment was made based on the median response (*Me; Q1–Q3*) of all study participants on a scale from 0 to 10. The ranking of the responses from the most important to the least important reason included: functional reasons—problems with biting, chewing, pronunciation (9 points), desire to improve appearance (8 points), need for replacement of old denture (5 points), willingness to take advantage of the possibility of replacing the prostheses every 5 years (0 points), another doctor’s recommendation (0 points), persuasion by relatives (0 points).

Problems with biting, chewing, and pronunciation were significantly more important for people with higher and secondary education than for people with primary and vocational education (higher education vs. primary, vocational education Me = 10; 7–10 vs. Me = 2.5; 0–10). For the remaining groups, it was not a statistically significant motivating factor for them to start prosthetic treatment (*p* > 0.05).

The willingness to replace the prostheses every 5 years was significantly more important for people with secondary education than for those with primary, vocational (Me = 7.5; 0–10 vs. 0; 0–8.25), and higher education (Me = 7.5; 0–10 vs. 0; 0–10). For the other groups, it was not a statistically significant motivating factor for them to undertake prosthetic treatment.

The remaining listed factors did not significantly influence the decision to use prosthetic treatment (*p* > 0.05).

### 3.4. Reasons for Undertaking Treatment at the UMP Prosthetics Clinic

Similarly, the assessment of motivating factors for starting treatment at the UMP Department of Prosthetics was made based on the median response (*Me; Q1–Q3*) of all participants’ responses on a scale from 0 to 10. The ranking of the responses from the most important to the least important included: the belief in the professionalism of doctors (10 points), reputation (10 points), modern equipment (10 points), quality of communication with the doctor (10 points), financial considerations (9 points), short waiting time (8 points), willingness to be treated by students (8 points), convenient access (6 points), recommendation by friends/relatives (5 points), referral from another doctor (0 points).

The belief in the professionalism of the doctors working in the Clinic was significantly more important (*p* < 0.05) for people aged 61–70 (Me = 10; 10–10), and over 70 (Me = 10; 9–10) than for people aged up to 40 (Me = 9; 5–10) and 51–60 (Me = 7; 0–10); for people with secondary education than for people with primary and vocational education (Me = 10; 9–10 vs. Me = 8; 0–10; *p* < 0.05); for economically inactive than for manual workers (Me = 10; 9–10 vs. Me = 9.5; 0–10; *p* < 0.05). For the remaining groups, it was not a statistically significant motivating factor for a patient to undertake prosthetic treatment (*p* > 0.05).

Recommendation by friends/relatives was significantly more important for those working manually than for the economically inactive (Me = 10; 0.25–10 vs. Me = 3; 0–10; *p* < 0.05). For other groups, it was not a statistically significant motivating factor for patients to undertake prosthetic treatment (*p* > 0.05).

Convenient access was significantly more important for those with young children than for those with adult children (Me = 10; 7.75–10 vs. Me = 5; 0–10; *p* < 0.05), and for those with just young children than for those with both young and adult children (Me = 10; 7.75–10 vs. Me = 0; 0–6.5; *p* < 0.05). In the remaining groups, it was not a statistically significant motivating factor for them to undertake prosthetic treatment.

Modern equipment was significantly more important for those with young children (Me = 10; 8.75–10) than for those who had no children at all (Me = 10; 7.5–10) and for those who had both young and adult children (Me = 2; 0–8.75; *p* < 0.05). For the other groups, it was not a statistically significant motivating factor for the patient to begin prosthetic treatment (*p* > 0.05).

Referral by another doctor was significantly more important for people aged up to 40 than for other patients (Me = 7; 1–8 vs. 0; 0–0; *p* < 0.05); for those working mentally than for inactive ones (Me = 2; 0–9.75 vs. 0; 0–0; *p* < 0.05); for those with young children than for those with adult children (Me = 2.5; 0–8.25 vs. 0; 0–0; *p* < 0.05); for those in need of a fixed prosthesis than for those in need of a complete prosthesis or two different types of prosthesis (0; 0–0 vs. 0; 0–4; *p* < 0.05). This was not a statistically significant motivating factor for undertaking prosthetic treatment in other groups (*p* > 0.05).

A short waiting time was significantly more important for people with primary and vocational education than for people with higher and secondary education (Me = 10; 7.25–10 vs. Me = 8; 0–10; Me = 6; 0–10; *p* < 0.05); for those with savings or investments compared to those without (Me = 8; 3–10 vs. Me = 5; 0–10; *p* < 0.05). For the remaining groups, it was not a statistically significant motivating factor for beginning prosthetic treatment (*p* > 0.05).

On the other hand, the quality of contact with the doctor was not statistically significant for any of the study groups (all *p* > 0.05).

The willingness to be treated by students was significantly more important for people with secondary education than for people with higher education (Me = 9; 5–10 vs. Me = 5; 0–8; *p* < 0.05); for people working outside the health service compared to those employed in the health service (Me = 8; 0–10 vs. Me = 0.5; 0–5.75; *p* < 0.05); as well as for people with savings or investments compared to those without them (Me = 8; 0–10 vs. Me = 6; 0–9; *p* < 0.05). In the remaining groups, it was not a statistically significant motivating factor for the undertaking of prosthetic treatment by patients (*p* > 0.05).

Financial considerations were significantly more important for people with secondary education than (Me = 10; 6.25–10 vs. Me = 8; 2–10; *p* < 0.05) for people with higher education; for people in a bad or average financial situation than for people in a good or very good situation (Me = 10; 8–10 vs. Me = 10; 5–10 vs. Me = 7.5; 0–10 *p* < 0.05); for people with a monthly income below 2000 PLN per person compared to respondents with an income above 2000 PLN (Me = 10; 8–10 vs. Me = 7; 2–10 *p* < 0.05); for people with savings or investments than for people without them (Me = 10; 5–10 vs. Me = 7; 0–10; *p* < 0.05). For the remaining groups, it was not a statistically significant motivating factor for patients to undertake prosthetic treatment (*p* > 0.05).

### 3.5. Reasons for Choosing Treatment Performed by Students

The most frequently given response was undertaking treatment due to its quality and professionalism—declared by 21 people, which constitutes 25.3% of respondents to this question. The second most frequent response was the low cost/economic reasons. This reason was important for 17 people, which is 20.48% of those responding to this question. The next two groups of answers, mentioned by 13 people (15.66%) each, are people who have been receiving treatment at the university for a long time, and people who, when undergoing treatment by students, give them the opportunity to learn and develop. For 12 people (14.45%), the reason was diligence, gentleness, meticulousness of the treatment carried out by the students of the treatment, and in one case, even the belief that people who are only just gaining experience, are able to do it more accurately. The quick admission date was the reason for 8 respondents, which is 9.63%, and for 8.43% of the respondents (7 people), the reason was the nice atmosphere in the classroom. Recommendation by relatives or friends was an important reason for 5 people, which is 6.02%. Four people (4.81%) gave the following answers, which could not be assigned to any of the above groups: good access to the clinic; referral by a dentist; the answers “students are more afraid than me” and “I like to experiment”.

**Figure 1:**
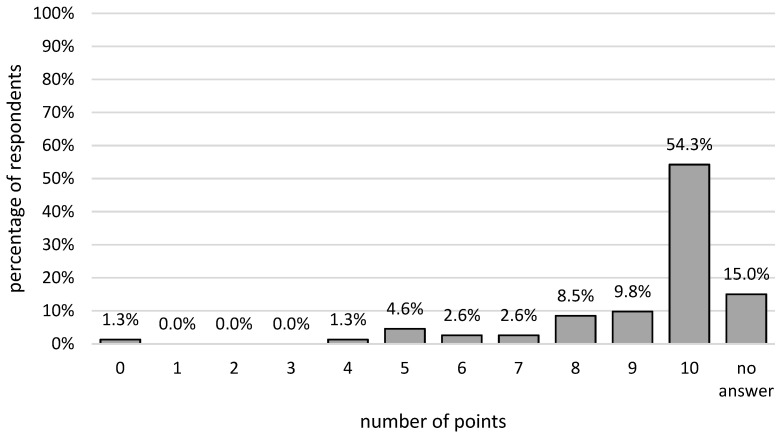
Percentage distribution of the evaluation of student treatment proficiency.

The assessment of the treatment professionalism of the students was significantly higher among people with primary, vocational (Me = 10; 10–10), and secondary (Me = 9; 9–10) education than among those with higher education (Me = 9; 6.5–10; *p* < 0.05); among people with savings or investments compared to those without them (Me = 10; 9–10 vs. Me = 9; 8–10; *p* < 0.05). For other groups, no statistically significant relationships were identified (*p* > 0.05). The highest mark was given out by 83 people. Fifteen people gave the students 9 points out of 10, while 13 gave them 8. Twenty-three participants did not respond to the question. The remaining 19 people evaluated student proficiency at a level equal to or lower than 7 (Figure 1).

**Figure 2:**
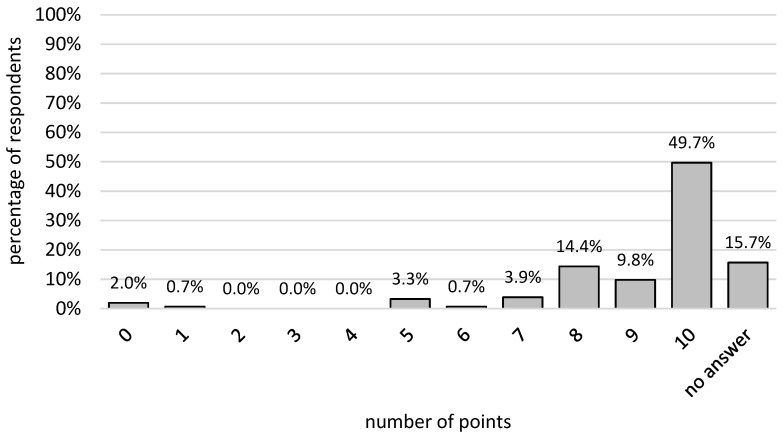
Percentage distribution of the evaluation of student communication skills.

The assessment of the communication skills of the students was significantly higher among people with primary, vocational, and secondary education than among people with higher education. For the remaining groups, no such statistically significant relationships were found (*p* > 0.05). Additionally, the assessment of the attitude of the students towards the patient was statistically insignificant for the respondents (*p* > 0.05). The highest number of points was given by 76 of the survey takers. Fifteen people evaluated communication skills at a level of 9. Twenty-two people graded this skill at a level of 8. Twenty-four respondents did not provide an answer to this question. The remaining 16 people marked answers between 0 and 7 points. The percentage distribution was presented in Figure 2.

### 3.6. Price List for Prosthetic Services

The price range for a ceramic crown on metal was from 800 PLN to 3500 PLN per point, averaging 1377.77 PLN. The commercial price at the UMP Prosthetics Clinic was 810 PLN, while the didactic price was 380 PLN.

An all-ceramic crown, depending on the variant on the market, costs in the range of 1250 PLN to 5000 PLN per point. The average cost is 2158.33 PLN. According to the price list in force at the Prosthetics Clinic of the UMP, for a crown made by a doctor, one must pay 1500 PLN, and a crown made during student classes costs 780 PLN.

For a cast partial denture in Poznan, a patient has to pay an average of 2160 PLN, and the price ranges from 1400 PLN to 4000 PLN. The commercial price at the UMP Prosthetics Clinic is 1600 PLN, while for patients treated by students it is 450 PLN for skeleton +25 PLN for each tooth.

The average market price for a complete prosthesis is 1589 PLN, and the prices range from 800 PLN to 3200 PLN. On the premises of UCSiMS, the commercial price is 1100 PLN, and the didactic price is 300 PLN for the jaw prosthesis and 350 PLN for the lower jaw prosthesis.

The market prices for non-detachable partial dentures range from 900 PLN to 2000 PLN, averaging 1150 PLN. The commercial price at the UMP Prosthetics Clinic, depending on the number of teeth in the denture, is 500 PLN (1–4 teeth), 750 PLN (5–8 teeth), and 1000 PLN (over 8 teeth), the didactic prices for the same tooth number are 350/450 PLN (Supplementary Materials).

## 4. Discussion

The issue of motivating factors for someone to undertake prosthetic treatment is much more complex than in the case of an interventionist patient, who seeks emergency care. Additionally, the clinical situation, socioeconomic factors, cultural conditions, previous experiences, or the opinion of others will influence motivation [23]. In reference to the motivating factors for a patient to undertake prosthetic treatment, in this study, the parameter rated the highest by the respondents was functional reasons—issues with biting, chewing, and pronunciation. Chewing and biting disorders can affect the condition of the whole organism. It was a statistically significant motivating factor for people with higher and secondary education than for people with primary and vocational education. Higher awareness of general health and prosthetic treatment options is perceived more by better-educated people. This is confirmed by the studies of Patil et al. [12]; Saha et al. [24]; Schützhold et al. [25]; and Baqar et al. [26]. Other researchers also observed that the desire to improve function and bite is the most important motivating factor for prosthetic treatment [13,27,28], and the results of this study and the study by Baqar et al. [26] showed no gender difference. In the study by Amjad and Aziz [29], 70.6% of men considered function as the most important reason for tooth replacement. In turn, in a study by Amine et al. [30], function was the main factor in each group of respondents, regardless of gender, age, income, and education level.

In the discussed study, the second most important factor was aesthetic considerations and the desire to improve appearance. None of the examined features has an impact on the assessment of the importance of the willingness to improve appearance, and it is equally important for all of them. However, other authors found some dependencies. In the study by Amjad and Aziz [29], the vast majority, as many as 83.03% of women, indicated external appearance as the main reason for treatment. Furthermore, in the studies by Mukatash et al. [31], a clear majority of respondents (over 80%) considered restoration of the front teeth more important than that of the posterior teeth, which is certainly related to aesthetics. Vieira et al. [32] utilized the theory of planned behavior to find out about intentions and behaviors influencing the willingness to undertake prosthetic treatment. They showed that the lack of a tooth or teeth in the maxilla and the anterior region had a significant impact on positive behavior towards prosthetic treatment.

Many patients find that the discomfort caused by tooth loss is greater when the missing tooth is visible, especially when they are in the company of strangers [33].

It is a well-known fact that the need for prosthetic treatment is related to the location of the missing tooth and the above-mentioned factors are most often given as having the greatest impact on starting treatment, depending more on the studied population, age, socioeconomic factors, or cultural conditions [34].

In our study, the third highest-rated factor was the need to replace the old prosthesis. In this parameter, the highest number of statistically significant relationships was detected.

The need to replace the old prosthesis was significantly more important for people aged over 70 than for people aged up to 40 and in the range of 41–50 years, as well as for people aged 51–60 and 61–70 years old than for people up to the age of 40. This dependency shows that older people need to replace dentures more than younger people because of their wear and tear. This is certainly due to the fact that the frequency of using removable dentures by patients increases with age [35,36]. In turn, in the study by Gârdan and Gârdan [6], people in the age range from 65 to 74 years old and over 75 years old constituted the largest group of people who did not consult a dentist. The authors considered this situation normal due to the high cost of prosthetic treatment, and the majority of people in this range are poor retirees [6].

The willingness to replace the prosthesis was also more important for people in an average financial situation than for people in a good or very good condition. This may be due to the fact that better-off people have more expensive restorations, which are naturally better, such as overdentures or permanent restorations, which wear out more slowly and require less frequent replacement. The literature shows that people with lower incomes use dental services less often and spend less money on them compared to people with higher income, even in subsidized health care systems [37,38].

Another significant difference was demonstrated between those in need of full dentures and those in need of partial or permanent dentures. The continuous process of reconstruction related to the aging of the body and the lack of bone stimulation through the periodontium causes the loss of retention and stabilization of the prosthesis with the passage of time of its use [39,40]. This can contribute to discomfort that lasts a long time and prevents you from continuing to function normally. In the case of partial dentures, despite the frequent loss of stabilization, the denture still has retention. As a result, patients, despite the significant mobility of the prosthesis on the base, do not feel as much discomfort resulting from the complete failure to maintain it, as in the case of full prostheses.

An additional look at this relationship is thrown by the fact that people in higher age groups require treatment with full dentures more often than younger people [41,42,43], which in turn is the same as the above results regarding age.

Functional considerations also turned out to be significantly more important for married people than for divorced people.

A similar motivator is the willingness to take advantage of the possibility of replacing the prosthesis every five years. This applies to people who had prostheses made under the National Health Fund (NFZ). According to the principles of reimbursement of services by the National Health Fund, each patient is entitled to a new prosthetic restoration every five years. Some patients use this privilege regardless of whether they feel the subjective need to replace the prosthesis due to the loss of its functional parameters or not. Such dentures are often replaced even if their parameters are maintained, and objectively stating there is no need to replace them, or it is enough to repair them by relining or rebasing.

The willingness to take advantage of the possibility of exchange was significantly more important for people with secondary education than for people with primary, vocational, and higher education. At this point, it is hard to find a relationship between the level of education and making decisions about replacing supplements. The dependence was demonstrated for people with both lower and higher education. The most likely explanation is that looking at individual parameters from socioeconomic data, the largest group were people with secondary education and people in the pre-retirement age. People in this age group are very often beneficiaries of NFZ services. Dental insurance systems have a positive impact on attitudes and motivation to use prosthetic treatment [44,45].

The respondents, when assessing the reasons for undertaking prosthetic treatment, considered referral by another doctor and persuasion by their relatives to be the least important. In both cases, no statistically significant relationships were found. Whereas Baqar et al. [26] showed in their study that friends and relatives were the main motivators of rehabilitation in 54.5% of respondents.

The main goal of this study, however, was to determine what factors influence the motivation to start treatment at the Prosthetics Clinic of the Poznan University of Medical Sciences, i.e., a place where patients are admitted as part of exercises, and the work is mainly performed by students.

The top-three-rated motivators are: belief in the professionalism of doctors; financial considerations; reputation.

Financial reasons came second in the question assessing motivation on a scale from 0 to 10. Moreover, in the open-ended question, economic reasons were the second most frequent answer. This reason was indicated by 17 people, which constitutes 20.48% of the answers given. However, it is precisely with this motivating factor that the most statistically significant relationships were demonstrated.

It was significantly more important for people with secondary education than for people with higher education. Among those surveyed, 86 people declared monthly income in the lowest range, also 86 people had secondary education. It can be presumed that people with higher education achieve a higher income, which means that the costs of treatment are not a significant reason for them to start therapy by students. The economic factor was significantly more important for people in a bad and average financial situation than for people who declared a good or very good financial condition. It seems obvious that people who subjectively assess their financial situation as worse will look for free or cheaper benefits more than people who assess their financial situation better.

Interestingly, in the studies of Leles et al. [46], conducted at the School of Dentistry of the Federal University of Goias (Goiania, Brazil), patients with the lowest income declared their economic status as average or good. Similarly, in the presented study, only 19.61% of the respondents described their financial income as bad, despite the fact that more than half of the respondents declared an income below 2000 PLN per person. For the latter, financial considerations were significantly more important than for the other groups. Economic factors and excessive costs were the main reasons for discontinuation or failure to undertake prosthetic treatment.

According to data from the Central Statistical Office (CSO) (2021a), the average salary in the first quarter of 2021 was 5681.56 PLN. On the other hand, the analysis of the number of benefits paid by Social Security after indexation in March 2021 shows that the highest percentage of pensions—18.8%—ranges from 1800.01 PLN to 2200.00 PLN. Thus, it can be seen that people who decided to undergo treatment in the Prosthetics Clinic are far below the average salary and, considering the age of the respondents and their professional activity, it can be seen that the group of patients with an income below 2000 PLN are mainly retirees and pensioners.

The above dependencies clearly show that people with the lowest financial status are treated in the Prosthetics Clinic of the Medical University of Warsaw for financial reasons. This may be partly due to the fact that the Clinic can provide services under the National Health Fund, but it is known that only a few procedures in the field of prosthetic treatment are reimbursed. However, among the respondents, 43.79% required treatment with partial dentures, probably some with skeletal dentures, and 25.49% required treatment with permanent dentures. Both the first and the second type are not reimbursed by the National Health Fund. Therefore, it can be presumed that patients who are mainly driven by economic factors when looking for a treatment facility, will choose the cheapest or one of the cheapest options.

In order to verify this thesis, the prices of the most frequently performed services during clinical exercises in the fourth and fifth year of studies in the field of medicine and dentistry were checked against the background of market prices in the Poznan area. The prices for the performance of a porcelain crown on a metal foundation, an all-ceramic crown, a complete denture, a partial settling denture, and a partial skeleton denture were considered.

The above data clearly show how low the costs of treatment provided by students are compared to commercial prices on the Poznan market. The same procedures performed within the same facility by doctors are over 100% more expensive, although their performance is outsourced to the same technical laboratory, and the same materials and equipment are used.

If these procedures were performed outside the University, the prices can be considered to be dumped, and when comparing them with the laboratory prices, it can be assumed that they cover only the costs incurred by UCSiMS. In this case, the additional “cost” borne by the patient is treatment by people who are just gaining their professional experience and working under the supervision of academic teachers. Therefore, individual visits take longer, and procedures often have to be repeated, which makes the treatment process significantly longer and less comfortable.

However, the results of the research show that the assessed professionalism of the treatment performed by students was quite high, as 72.55% of patients rated it in the range from 8 to 10; moreover, this reason was the most frequently given answer in the open question (25.3%).

The explanation for these seemingly contradictory facts may be that the most important motivating factor for a patient to start treatment in the Prosthodontics Clinic of the Medical University of Warsaw was the conviction about the professionalism of doctors, and then only financial considerations.

Therefore, it can be concluded that patients deciding to undergo treatment by students, which, despite possible drawbacks, is constantly supervised by doctors/academic teachers who are often specialists with scientific degrees/titles, is still considered professional and performed at the highest level.

However, this belief is important only for people with primary, vocational, and secondary education.

In addition, the motivating factors that can be associated with the expected professionalism of treatment in the university unit are reputation and the quality of contact with the doctor. However, no significant relationships between them were found.

The willingness to be treated by students was significantly more important for people with secondary education than for people with higher education and for people working outside the health service. The first correlation confirms the abovementioned belief in the professionalism of treatment carried out by students in people without higher education. The fact of the latter relationship seems interesting, which shows that medical professionals do not want to be treated by students. It can be presumed that knowing the process of teaching, and gaining experience from an autopsy, they would not want to be treated by themselves at the beginning of this path, which seems to be a logical justification. In addition, the research of Mukatash et al. clearly indicates that doctors and paramedics were more aware of their needs and expectations for prosthetic treatment than the rest of society [31]. The answers to the open question provide clear evidence that some patients treat being treated by students as a privilege. Twelve people (14.45%) indicated diligence, gentleness, and meticulousness as the reason for the treatment by students, with one even believing that people who are just learning are able to do it more accurately. Seven people (8.43%) mentioned the pleasant atmosphere in the classroom as the reason, while 13 people (15.66%) wanted to undergo treatment by students so that they could gain experience (altruistic reasons). In addition, 13 people (15.66%) have been receiving treatment from students for a long time, which means that they have had positive experiences since they continue to use their services. Thus, a total of 45 out of 83 (54.21%) responses to the question in which patients had complete freedom, gave a positive response to the treatment by students.

The very experience of communing with students was assessed very positively by the patients.

The ability of students to communicate was assessed equally highly. Additionally, this assessment was significantly higher among people with primary, vocational, and secondary education than among people with higher education, which is more proof that less educated people are more enthusiastic about treatment by students. It can be argued that better-educated people, due to higher expectations of treatment, are less willing to undergo treatment by students.

Among the remaining motivating factors, which are no longer directly related to the students themselves, the following were rated relatively high: the possibility of using modern equipment and short waiting times. Although some statistically significant relationships were demonstrated for both parameters, they are difficult to interpret.

Modern equipment was significantly more important for those with young children and no children than for those with young and adult children. An attempt can be made to link having younger children and the lack thereof with the lower age of the patients. In turn, younger people can pay more attention to the presence of modern technological equipment and the appearance of the office. These conclusions are indirectly related to the results of Gârdan and Gârdan [6], who showed that younger people pay more attention to office hygiene than people in other age groups.

On the other hand, the short waiting time was statistically more important for people with primary and vocational education than for people with higher and secondary education, and people with savings or investments.

Convenient access was significantly more important for those with young children than for those with adult children and those with both young and adult children. Having young children requires good time planning, therefore convenient commuting is a significant facilitation; moreover, having younger children can again be associated with the lower age of their parents. In a study by Gârdan and Gârdan [6], lack of time was the second most common reason for not undertaking dental treatment in the 25 to 34 and 35 to 49 age groups.

The influence of relatives on starting treatment at the UMP Prosthetics Clinic was higher than in the case of their influence on starting prosthetic treatment in general. Additionally, their influence on motivation was significantly more important for those working physically than for the economically inactive. Gârdan and Gârdan [6] showed a correlation between the advice of friends and relatives and age. When choosing an office, younger people consider the opinion of their relatives more than older people.

The lowest rated motivating factor in this study was referral by another doctor and it was significantly more important for people up to 40 years of age than for other patients. The remaining relationships were significantly more important for those working mentally than for the economically inactive, for those who had young children than for those who had adult children, and for those in need of a fixed prosthesis than for those who needed a complete prosthesis or two different types of prosthesis. The last three relationships can be related to age.

Although this study did not investigate the relationship between the type of missing teeth and the place of residence or other socioeconomic factors, the results indicate that most of the respondents lived in urban areas, and most people required treatment with removable, partial, or full dentures. Zitzmann et al., in their research, showed that in Europe, removable dentures are more often used in rural areas than in cities [47]. On the other hand, the results of the research conducted by Øzhayat et al. [33] indicate differences in the treatment approach depending on the place of residence. People living in rural areas showed greater acceptance of removable restorations than patients in urban areas. The latter considered implant prosthetic solutions to be the best.

An interesting relationship was also observed between studies conducted in other prosthetic clinics in different countries. Namely, in most of the cited results, the majority of the research group consisted of women. Similarly, in this study, women accounted for 66.67% of the respondents. In a study conducted at the Department of Prosthodontics (Khyber College of Dentistry) Peshawar, 61% were women [48]. In the study by Kulczyńska et al. [49] at the Prosthetics and Implantoprosthetics Clinic of the Specialist Dental Center of the Medical University of Gdansk, women accounted for 60.31%. In a study by Amine et al. [30] at the Prosthetic Department of Casablanca’s Dental Consultation and Treatment Center, women constituted 60% of the respondents. Various hypotheses appear in literature trying to explain this tendency. Life expectancy increased on average by 6.2 years compared to 1990, lengthening to almost 72 years in 2013. Among women, the increase in life expectancy was slightly higher (6.6 years) than among men (5.8 years) [26], which also resulted in an increase in the prosthetic needs of women compared with men. In turn, the higher rate of toothlessness can be explained by the different treatment patterns of the two genders. Women more often than men use medical services, including dental treatment. Moreover, women are more likely to remove single remaining teeth for aesthetic reasons to replace them with dentures However, Khalifa et al. reported increased tooth loss in women combined with low socioeconomic status and low education levels [50].

An extremely important element accompanying the creation of questionnaires is their validation, i.e., the assessment of suitability, validity, and accuracy. First of all, it is important in the case of questionnaires translated from a foreign language. It is not only about the fidelity of the translation, but also about cultural adaptation, which is an inseparable element of validation. It must meet the following criteria: fidelity to the translation of the questionnaire into Polish, the criterion of functional equivalence, the principle of façade equivalence, and the criterion of fidelity of the reconstruction [51]. Although our proprietary questionnaire was not borrowed from other available questionnaires, its validation should be considered.

Although there are studies of the motivation, needs, and expectations of prosthetic patients in various university units, none of the works cited deal with the problem of treating patients by students or during student classes. This fact can be taken as a strong point of this study because it touches on the problem of patients’ motivation a bit deeper and delimits the motivation for prosthetic treatment in general, and for treatment carried out in a specific unit, characterized by such specificity as it is. This work precisely describes the motivating factors in the group of patients of the UMP Prosthetics Clinic. At the same time, it becomes a limitation in the knowledge of population factors that lead to prosthetic therapy. The above results should be compared with studies carried out in other units, including the private sector.

For many social groups, the costs of prosthetic treatment are still the main barrier to starting therapy. It was believed that the low cost of treatment offered during student classes would be the main factor motivating patients to start treatment at the UMP Prosthetics Clinic. However, it turns out, that the authority of the university unit is of greater importance, and patients undertake treatment because of the belief in its professionalism. This belief depends on the education level. Additionally, some patients undergo treatment by students because they can see positive aspects in it, both for them and for students. Hence, the evaluation of the treatment provided by students, as well as their communication skills and their attitude, are rated very highly, although, again, the evaluation is influenced by the education level. Based on the analysis of sociodemographic data, it can be said that a statistical patient undertaking treatment at the Department of Prosthetics of the Poznan University of Medical Sciences will be a retiree/pensioner.

## 5. Conclusions

The main motivating factors for patients to undertake prosthetic treatment are functional and aesthetic considerations, which are confirmed by the results of the conducted research and a literature review.

## Figures and Tables

**Table 1:** Average level of the need to replace old prostheses (scale of 0–10) due to selected parameters in sociodemographic groups.

Parameter	Group	Number of Points			*p*-Value ^1^
		Me *p*-Value ^2^	Q1	Q2	
Age groups [years]	Up to 40 years old (N = 17)—A	0 ^C,D,E^	0	0	*p* = 0.004
	41–50 years old (N = 32)—B	0 ^E^	0	10	
	51–60 years old (N = 25)—C	8 ^A^	0	10	
	61–70 years old (N = 46)—D	9 ^A^	0	10	
	Above 70 years old (N = 33)—E	10 ^A,B^	0	10	
Occupational activity	Physical work (N = 34)—A	1	0	10	*p* = 0.03
	Mental work (N = 30)—B	0 ^B^	0	8	
	Economic inactivity (N = 89)—C	9 ^C^	0	10	
Marital status	Bachelor/maiden (N = 27)—A	0 ^D^	0	10	*p* < 0.001
	Married (N = 78)—B	6 ^C,D^	0	10	
	Divorced (N = 25)—C	0 ^B,D^	0	8	
	Widower/widow (N = 23)—D	10 ^A,B,C^	9	10	
Children	None (N = 20)—A	0	0	9.25	*p* = 0.04
	Young (N = 16)—B	0 ^C^	0	6	
	Adult (N = 103)—C	9 ^B^	0	10	
	Young and adult (N = 14)—D	0	0	9.5	
Financial status	Bad (N = 30)—A	0	0	10	*p* = 0.02
	Average (N = 73)—B	9 ^C^	9	10	
	Good, very good (N = 48)—C	0 ^B^	0	10	
Type of treatment	Fixed denture (N = 39)—A	0 ^C^	0	9.5	*p* = 0.008
	Partial denture (N = 67)—B	0 ^C^	0	10	
	Complete denture (N = 30)—C	9.5 ^A,B^	7.25	10	
	Various (N = 12)—D	7.5	0	10	

Data presented by median (*Me*) with quartiles (*Q1*—lower quartile, *Q3*—upper quartile); *p*-value ^1^—statistically significant differences between groups by the Kruskal-Wallis’s test; *p*-value ^2^—the detection of statistically significant differences—post-hoc analysis by the Dunn’s test.

## Data Availability

The data used to support the findings of this study are included in the article. The data will not be shared due to third-party rights and commercial confidentiality.

## Supplementary Materials

The following supporting information can be downloaded at: https://www.mdpi.com/article/10.3390/ijerph19095703/s1, Word File S1: Questionnaire for patients being treated at the dental prosthetics clinic of the Poznan University of Medical Sciences.
