# Supplementary material for: Assessment of the Socioeconomic Status and Analysis of the Factors Motivating Patients to Apply for Prosthetic Treatment by Students of Dentistry at the Poznań University of Medical Sciences
Source: Int J Environ Res Public Health. 2022 May 7;19(9):5703. doi: 10.3390/ijerph19095703 (PMC9104844; doi:10.3390/ijerph19095703)
Supplement: Supplementary file 1 [file ijerph-19-05703-s001.zip › ijerph-1667219-supplementary.pdf]

Supplementary Materials

Questionnaire

QUESTIONNAIRE FOR PATIENTS BEING TREATED AT THE DENTAL PROSTHETICS CLINIC OF THE POZNAN UNIVERSITY OF MEDICAL SCIENCES

The following questionnaire is anonymous, its aim is to assess the motivations for patients to undertake prosthetic treatment at the Dental Prosthetics Clinic of the Poznan University of Medical Sciences. Its' results will be used for research purposes only. Please select the best-suited answer.

1. **What is your age?…………**

2. **Please select your sex.**

   Female

   Male

3. **What is your education level?**

   Primary

   Vocational

   Secondary

   Higher

4. **What kind of professional work do you do?**

   Physical *(e.g., construction worker, electrician, craftsman)*

   Mental *(office worker, clerk, teacher, accountant)*

   I am a retiree/pensioner

   I'm not working

5. **Are you employed in the health service?**

   Yes

   No

6. **What is your place of residence?**

City above 100 thousand inhabitants

Town between 50-100 thousand inhabitants

Town between 10-50 thousand inhabitants

Town between 5-10 thousand inhabitants

Town up to 5 thousand inhabitants

Village/small town neighboring a large town

Village

7. **What is your marital status?**

Bachelor/maiden

Married

Divorced

Widow/widower

Single

8. **Do you have children?**

I do not have children

Yes, I have young children

Yes, I have adult children

9. **What is your financial status?**

Bad

Good

Average

Very good

10. **What is your monthly household income?**

    Up to 2 thousand PLN per person

    Between 2 and 5 thousand PLN per person

    Above 5 thousand PLN per person

11. **Do you have savings/investments?**

    NO

    YES

If yes, please indicate in what form:

Cash savings above 10 thousand PLN

Deposit

Real estate

Stocks

Works of art

Other………………………

12. **What is the main reason why you have decided to undertake prosthetic treatment***? Please assess your motivation on a scale of 0 to 10 in each of the subsections, from a to f, where 0 indicates no influence on making the decision, and 10 indicates a very strong influence.*

    a) **Old dentures and the need to replace them (uncomfortable, not aesthetic, worn)**

    0 – 1– 2– 3– 4 – 5– 6– 7–8 – 9 – 10

    b) **Aesthetic reasons– the desire to improve appearance**

    0 – 1– 2– 3– 4 – 5– 6– 7–8 – 9 – 10

    c) **Functional reasons – problem with biting, chewing, pronunciation**

    0 – 1– 2– 3– 4 – 5– 6– 7–8 – 9 – 10

    d) **Treatment prescribed by another doctor**

    0 – 1– 2– 3– 4 – 5– 6– 7–8 – 9 – 10

    e) **Encouragement to undergo treatment by relatives (husband/wife, others)**

0 – 1– 2– 3– 4 – 5– 6– 7–8 – 9 – 10

f) It is possible to replace dentures every 5 years, so I want to take advantage of it

0 – 1– 2– 3– 4 – 5– 6– 7–8 – 9 – 10

13. **What was the main reason for the decision to undertake treatment at the Dental Prosthetics of the UMP?** *Please assess your motivation on a scale of 0 to 10 in each of the subsections, from a to f, where 0 indicates no influence on making the decision, and 10 indicates a very strong influence.*

a) My belief in the professionalism of the doctors working in the Clinic

0 – 1– 2– 3– 4 – 5– 6– 7–8 – 9 – 10

b) Reputation

0 – 1– 2– 3– 4 – 5– 6– 7–8 – 9 – 10

c) Recommendation by friends/relatives

0 – 1– 2– 3– 4 – 5– 6– 7–8 – 9 – 10

d) Convenient access

0 – 1– 2– 3– 4 – 5– 6– 7–8 – 9 – 10

e) The possibility of using modern equipment

0 – 1– 2– 3– 4 – 5– 6– 7–8 – 9 – 10

f) Referral by another doctor

0 – 1– 2– 3– 4 – 5– 6– 7–8 – 9 – 10

g) Short waiting time for the visit

0 – 1– 2– 3– 4 – 5– 6– 7–8 – 9 – 10

h) Quality of contact with the doctor

0 – 1– 2– 3– 4 – 5– 6– 7–8 – 9 – 10

i) Willingness to be treated by dentistry students

0 – 1– 2– 3– 4 – 5– 6– 7–8 – 9 – 10

j) Financial considerations

0 – 1– 2– 3– 4 – 5– 6– 7–8 – 9 – 10

14. How do you rate the professionalism of the treatment provided by students on a scale from 0 to 10?

Very bad 0 – 1– 2– 3– 4 – 5– 6– 7–8 – 9 – 10*Very good*

15. How do you rate the students' attitude towards the patient on a scale from 0 to 10?

Very bad 0 – 1– 2– 3– 4 – 5– 6– 7–8 – 9 – 10*Very good*

16. How do you rate the ability of the students to answer your questions (communication) on a scale from 0 to 10?

Very bad 0 – 1– 2– 3– 4 – 5– 6– 7–8 – 9 – 10*Very good*

17. What kind of prosthetic treatment do you expect or are in the process of receiving?

Fixed denture (crown, bridge, root post, veneer)

Partial dentures (removable denture – acrylic, skeletal – in the presence of teeth)

Complete denture (in case of complete absence of teeth)

18. Why did you choose treatment provided by dentistry students?

……………………………………………………………………………………

List of treatment prices on the Poznan market

	All-ceramic crown	Porcelain crown on metal	Complete denture	Partial denture	Skeletal prosthesis
Treatment room 1	1500 PLN	1200 PLN – 1400 PLN	1800 PLN	1000 PLN – 2000 PLN	2000 PLN
Treatment room 2	1600 PLN	950 PLN	900 PLN	1000 PLN	1800 PLN
Treatment room 3	1500 PLN	900 PLN	1000 PLN	900 PLN	1700 PLN
Treatment room 4	1700 PLN	1200 PLN	900 PLN	900 PLN	1400 PLN
Treatment room 5	5000 PLN	3500 PLN – 5000PLN	-	-	-
Treatment room 6	1950 PLN	1450 PLN	2500 PLN	1700 PLN	2000 PLN
Treatment room 7	2600 PLN	-	3200 PLN	-	4000 PLN - 8000 PLN
Treatment room 8	2500-2900 PLN	-	-	-	-
Treatment room 9	2700 PLN	1500 PLN	1900 PLN	-	2500 PLN
Treatment room 10	1600 PLN	900 PLN	2000 PLN	1500 PLN	1800 PLN
Treatment room 12	1250 PLN	800 PLN	800 PLN – 950 PLN	-	1400 PLN
UCSiMS (commercial)	1500 PLN	830 PLN	1100 PLN	500 PLN – 1000 PLN	1600 PLN
UCSiMS (didactic)	780 PLN	380 PLN	350 PLN jaw<br>300 PLN lower jaw	350 – 450 PLN	450 + 25PLN/tooth

The prices given in the table are derived from the price lists available on the websites of individual institutions.
